# Does Thrombosis Play a Causal Role in Lacunar Stroke and Cerebral Small Vessel Disease?

**DOI:** 10.1161/STROKEAHA.123.044937

**Published:** 2024-03-26

**Authors:** Fatemeh Koohi, Eric L. Harshfield, Alexey Shatunov, Hugh S. Markus

**Affiliations:** Department of Clinical Neurosciences, Stroke Research Group, University of Cambridge, United Kingdom.

**Keywords:** arteries, cerebral small vessel diseases, stroke, lacunar, thromboembolism, thrombosis

## Abstract

**BACKGROUND:**

The importance of thromboembolism in the pathogenesis of lacunar stroke (LS), resulting from cerebral small vessel disease (cSVD), is debated, and although antiplatelets are widely used in secondary prevention after LS, there is limited trial evidence from well-subtyped patients to support this approach. We sought to evaluate whether altered anticoagulation plays a causal role in LS and cSVD using 2-sample Mendelian randomization.

**METHODS:**

From a recent genome-wide association study (n=81 190), we used 119 genetic variants associated with venous thrombosis at genome-wide significance (*P*<5*10^−8^) and with a linkage disequilibrium r^2^<0.001 as instrumental variables. We also used genetic associations with stroke from the GIGASTROKE consortium (62 100 ischemic stroke cases: 10 804 cardioembolic stroke, 6399 large-artery stroke, and 6811 LS). In view of the lower specificity for LS with the CT-based phenotyping mainly used in GIGASTROKE, we also used data from patients with magnetic resonance imaging–confirmed LS (n=3199). We also investigated associations with more chronic magnetic resonance imaging features of cSVD, namely, white matter hyperintensities (n=37 355) and diffusion tensor imaging metrics (n=36 533).

**RESULTS:**

Mendelian randomization analyses showed that genetic predisposition to venous thrombosis was associated with an increased odds of any ischemic stroke (odds ratio [OR], 1.19 [95% CI, 1.13–1.26]), cardioembolic stroke (OR, 1.32 [95% CI, 1.21–1.45]), and large-artery stroke (OR, 1.41 [95% CI, 1.26–1.57]) but not with LS (OR, 1.07 [95% CI, 0.99–1.17]) in GIGASTROKE. Similar results were found for magnetic resonance imaging–confirmed LS (OR, 0.94 [95% CI, 0.81–1.09]). Genetically predicted risk of venous thrombosis was not associated with imaging markers of cSVD.

**CONCLUSIONS:**

These findings suggest that altered thrombosis plays a role in the risk of cardioembolic and large-artery stroke but is not a causal risk factor for LS or imaging markers of cSVD. This raises the possibility that antithrombotic medication may be less effective in cSVD and underscores the necessity for further trials in well-subtyped cohorts with LS to evaluate the efficacy of different antithrombotic regimens in LS.

Lacunar stroke (LS), also known as small artery occlusion, accounts for up to a quarter of all ischemic strokes.^1^ It is typically caused by cerebral small vessel disease (cSVD), which affects the small perforating or penetrating arteries within the white matter and deep gray matter nuclei.^1^ cSVD is a major cause of nontraumatic intracerebral hemorrhage, vascular dementia, and cognitive impairment.^1^ Despite its importance, there are few proven treatments for cSVD, and a major factor leading to this is a limited understanding of the underlying disease mechanisms.^2,3^

**See related article, p 943**

Thromboembolic therapy with antiplatelet agents and anticoagulants is a mainstay of secondary prevention for ischemic stroke. Thromboembolism plays a key role in the pathogenesis of cardioembolic stroke and large-artery stroke. However, whether thrombosis is an initiating event in LS is uncertain.^3^ Large randomized controlled trials have shown that antiplatelet agents reduce the risk of recurrent stroke, but these trials have mainly examined overall stroke, and the stroke subtyping available within the trials does not enable robust estimates of their efficacy for LS.^4^ While one secondary analysis of a randomized controlled trial suggested efficacy for LS,^5^ another study showed that dual antiplatelet therapy did not result in a reduction in ischemic events compared with single antiplatelet therapy but did increase hemorrhagic complications.^6^ This risk of intracerebral hemorrhage is particularly important as cSVD has been shown to be an important underlying pathophysiological mechanism leading to intracerebral hemorrhage,^7^ especially when patients are treated with antithrombotic medications.^8–10^ This consideration is even more relevant with the increasing use of dual antiplatelets to reduce the risk of early recurrent stroke.^11^

One way of examining whether 2 processes are causally related is Mendelian randomization (MR), which uses genetic variants as instrumental variables to evaluate whether there is likely to be a causal effect.^12^ Because genetic variants are randomly allocated during meiosis, MR is less influenced by reverse causality and confounding and can yield results equivalent to those of randomized controlled trials.^13^ Previous genetic studies have suggested that thrombotic mechanisms are less important for cSVD compared with other stroke subtypes.^14,15^ A recent genome-wide association study (GWAS) performed in venous thrombosis has provided a much more comprehensive evaluation of genetic factors associated with thrombosis risk and, therefore, more power to examine associations with LS and cSVD. We utilized GWAS summary statistics from this study in an MR analysis to comprehensively investigate whether venous thrombosis is a causal risk factor for both LS and imaging markers of cSVD. It has been suggested that LS may have distinct subtypes, each with a different pathophysiology,^16,17^ one associated with atheroma at the origins of the perforating arteries, and the other with a diffuse small vessel arteriopathy. Therefore, to increase confidence in the findings, we included a cohort of magnetic resonance imaging (MRI)–confirmed LS in addition to a larger cohort of clinically defined LS.

## METHODS

### Data Availability

Data from the UK Biobank are available through the application at http://www.ukbiobank.ac.uk/using-the-resource/. Individual-level data from the NINDS Stroke Genetics Network Study are available to researchers through the Database of Genotypes and Phenotypes. The summary statistics from the Cohorts for Heart and Aging Research in the Genomic Epidemiology Consortium can be obtained directly from dbGaP (https://www.ncbi.nlm.nih.gov/gap/). The GWAS meta-analysis summary statistics for venous thromboembolism (VTE) are publicly available at https://www.decode.com/summarydata, and the summary statistics for stroke from the GIGASTROKE Consortium can be obtained from the GWAS Catalog (https://www.ebi.ac.uk/gwas/; study accession numbers: GCST90104534–GCST90104563).

### Study Design and Data Sources

#### Genetic Associations With Venous Thrombosis

We obtained genetic variants strongly associated with venous thrombosis from the largest and latest GWAS of VTE, which used 6 data sets (including CHB-CVDC/DBDS, UK Biobank, FinnGen Freeze, deCODE, Intermountain Healthcare, and MVP) with a total of 81 190 cases of VTE and 1 419 671 controls.^18^ VTE cases were defined based on hospital records in all studies, while UK Biobank included both self-reported cases and hospital episodes.^18^

#### Genetic Associations With Stroke

We studied associations with LS and other stroke subtypes from the GIGASTROKE Consortium, in which LS subtyping was performed largely with computed tomography (CT) imaging. In view of the lower specificity of LS subtyping using CT, we also studied a second cohort with MRI-confirmed LS.

##### All Stroke, Ischemic Stroke, and Ischemic Stroke Subtypes

We obtained summary statistics for genetic associations with stroke subtypes from the GIGASTROKE Consortium,^19^ consisting of 73 652 patients with stroke and 1 234 808 controls of European ancestry. The summary statistics were available for 73 652 cases with any stroke (AS), 62 100 cases with any ischemic stroke (AIS), 10 804 cases with cardioembolic stroke (CES), 6399 cases with large-artery stroke (LAS), and 6811 cases with LS. Stroke cases were defined based on WHO criteria, and ischemic stroke subtypes were classified according to the TOAST (Trial of ORG 10172 in Acute Stroke Treatment) criteria.^20^

##### MRI-Confirmed LS

The majority of cases in the GIGASTROKE data set were subtyped using CT, which has only a modest level of accuracy,^21,22^ but it has been shown that by using MRI, the improved specificity of stroke subtyping can increase the power to detect significant genetic associations with LS.^23^ Therefore, we conducted a second analysis of the association with LS using GWAS data from an MRI-confirmed cohort of patients with LS. Furthermore, to allow us to examine associations with subtypes of LS, the LS cases were further categorized into multiple lacunar infarcts with leukoaraiosis and isolated lacunar infarcts, as previously described,^17,24^ and we conducted additional separate analyses for each subtype. Leukoaraiosis was defined as having confluent white matter hyperintensities (WMHs) on MRI, that is, a grade of 2 or higher on the semiquantitative Fazekas scale.^25^

For the GWAS of MRI-confirmed cases, we used previously published data from the UK DNA LS studies 1 and 2 and other studies within the International Stroke Genetics Consortium.^23^ We reanalyzed these data with the addition of 690 cases from DNA lacunar 2 and 31 cases from the Apathy After Stroke Study, a prospective clinical cohort study aiming to follow-up ≈200 patients with stroke from the time of their stroke to a year after. Furthermore, we excluded 375 patients of non-European ancestry.

The genetic data from these studies were imputed against the GRCh38-build reference panel using the TOPMed imputation server^26–28^ and combined in a single data set. For the genetic quality control, we used algorithms implemented in PLINK, versions 1.9 and 2.^29^ Further details are provided in Supplemental Methods and Table S1.

#### Genetic Associations With Imaging Markers of cSVD

We also examined associations with MRI markers of cSVD, WMH volume, and diffusion tensor imaging (DTI) metrics. We updated a previous GWAS^30^ on both WMH and DTI metrics of white matter tracts, including mean diffusivity (MD, the degree of diffusion), fractional anisotropy (the directionality of diffusion), and peak width of skeletonized MD (PSMD, an automated measure based on skeletonization and histogram analysis). This previous GWAS included cases from the UK Biobank, Cohorts for Heart and Aging Research in Genomic Epidemiology, and a UK hospital-based study.^30^ The data from the Cohorts for Heart and Aging Research in Genomic Epidemiology Consortium,^31,32^ which was accessed through the Database of Genotypes and Phenotypes (Database of Genotypes and Phenotypes; study: phs000930.v10.p1), involved a multiethnic population of 21 079 individuals without dementia or stroke at baseline, of European (n=17 936), African (n=1943), Hispanic (n=795), and Asian ancestry (n=405). It included WMH metrics but not DTI metrics. We focused exclusively on the data from individuals of European ancestry (n=17 936). We updated the previously published analysis^30^ to include an additional 18 972 participants from the UK Biobank with MRI and DTI measurements that were recently made available.

Full details of the UK Biobank study design and population have been provided elsewhere.^33^ Briefly, the UK Biobank is a large, population-based cohort study including ≈500 000 participants aged 40 to 69 years recruited from across Great Britain between 2006 and 2010. Starting in 2014, a subset of 100 000 participants began to undergo brain MRI examinations after the initial assessment.^34^ The UK Biobank MRI acquisition protocol and pipeline for the production of imaging-derived phenotypes have been described in more detail elsewhere.^35^ In this study, we used the UK Biobank imaging data on ≈45 000 individuals released in April 2023. We derived PSMD from the original DTI-MRI scans.^36^

We utilized genotype data that was imputed to the Haplotype Reference Consortium panel and made available by UK Biobank in June 2017. The imputation and quality control procedures used in the UK Biobank study are detailed elsewhere.^37^ Further details are provided in Supplemental Methods and Table S2.

### Statistical Analyses

#### Genome-Wide Association Analyses

The association analyses between allele dosages and MRI-confirmed LS, as well as imaging markers of cSVD, were performed using REGENIE (version 3.2.8), which efficiently handles binary traits with unbalanced case-control ratios, population structure, and relatedness, and can estimate statistics for multiple phenotypes simultaneously.^38^ Logistic regression with firth (Firth likelihood ratio test) and loocv (leave-one-chromosome-out cross-validation) options was used for MRI_LS (n=3199), multiple lacunar infarcts with leukoaraiosis (n=1658), and isolated lacunar infarct (n=1133), with sex and the first 6 principal components as covariates. Linear regression was used for WMH (n=37 355), fractional anisotropy (n=36 533), MD (n=36 460), and PSMD (n=36 012), with adjustment for age at MRI, sex, genotyping array, the UK Biobank imaging assessment center, the first 10 principal components for genetic ancestry, mean task functional MRI head motion, and mean resting-state functional MRI head motion. Missing values for task functional MRI and resting-state functional MRI were imputed using the predictive mean matching method based on all covariates, utilizing the mice package in R. A *Z*-score-based meta-analysis was conducted using the METAL tool^39^ to combine the GWAS results for WMH from the UK Biobank with those from the Cohorts for Heart and Aging Research in Genomic Epidemiology Consortium.

#### MR Analyses

To estimate the causal effects of venous thrombosis on each stroke subtype, we used a 2-sample MR that utilizes summary association results estimated in 2 independent studies and allows more statistical power to infer causal relationships.^40^ MR is a form of instrumental variable analysis that uses genetic variants associated with exposure as instrumental variables. The validity of MR relies on 3 main assumptions: (1) the IVs must be associated with the exposure (the relevance assumption), (2) the IVs must not be associated with any confounders that affect exposure or outcome (the independence assumption), and (3) the IVs must be associated with the outcome only through exposure and not through any other independent pathway (the exclusion restriction assumption).^40^ The third assumption is generally the most challenging and requires the absence of horizontal pleiotropy.^40^

For the exposure IVs, we initially selected genetic variants that were significantly associated with the risk of VTE at *P*<5*10^−8^ in the summary statistics from the latest GWAS of VTE, which ensured the validity of the first MR assumption. We then performed clumping at a 1000-kb window to select only independent IVs with a linkage disequilibrium threshold of r^2^<0.001 to reduce the likelihood of bias.

Furthermore, we ensured that the associations of genetic variants with venous thrombosis and each stroke subtype were expressed with respect to the same effect allele by harmonizing the association results in the exposure and outcome data sets. We then assessed the strength of IVs by calculating the F-statistic for each variant, using the approximation method described by Bowden et al,^41^ which involves dividing the square of the effect of the variant on the exposure by the variance of its effect on the exposure.

We used the inverse-variance weighted method with random effects as our primary analysis method, which involves a meta-analysis of causal estimates from individual genetic instruments to yield a single estimate of the causal effect of coagulation on each stroke subtype.^40^ To assess the consistency of causal estimates, we also used other robust MR methods, including MR-Egger, which estimates unmeasured pleiotropy,^42^ and a weighted median-based method that evaluates the stability of strong variant-outcome relationships.^43^ To assess evidence of pleiotropic effects statistically, we conducted sensitivity analyses using MR-Egger regression and outlier-corrected MR-PRESSO (Mendelian Randomization Pleiotropy Residual Sum and Outlier) tests. The heterogeneity between IVs was also checked by the Cochran Q test. Furthermore, the MR-Steiger test was performed to infer the true direction of causality. We further conducted a leave-one-out analysis that reestimates the effect by sequentially excluding one variant at a time to assess the sensitivity of the MR estimates to individual variants that might have a horizontal pleiotropic effect.

All statistical analyses were performed in R 4.3.0 (R Core Team, 2021). The standardization of GWAS summary statistics was conducted using the MungeSumstats package, version 1.8.0, and MR analyses were performed using the packages TwoSampleMR, version 0.5.4, and MRPRESSO, version 1.0. To address the issue of multiple testing, a false discovery rate–adjusted *P*<0.05 was used to identify significant associations. This study is reported as per the guidelines for the STROBE-MR checklist (Strengthening the Reporting of Observational Studies in Epidemiology Using Mendelian Randomization).^44^

## RESULTS

### Genetic Markers of Thrombosis

A total of 10 468 genetic variants associated with VTE of genome-wide significance (*P*<5*10^−8^) were obtained. Out of these, 10 349 variants were excluded either due to their absence from the linkage disequilibrium reference panel or high linkage disequilibrium (r^2^>0.001), giving 119 independent variants that were used as IVs of thrombosis (Table S3). Some variants were further excluded during the harmonization step because they were either palindromic or not present in the outcome data sets. As a result, the final analysis for each outcome utilized a varying number of IVs, ranging from 69 to 113 (Table). The average F-statistic of the IVs for venous thrombosis ranged from 101.5 to 111.2 for each specific outcome, suggesting that the IVs are unlikely to be susceptible to weak instrument bias (Table).

**Table.**
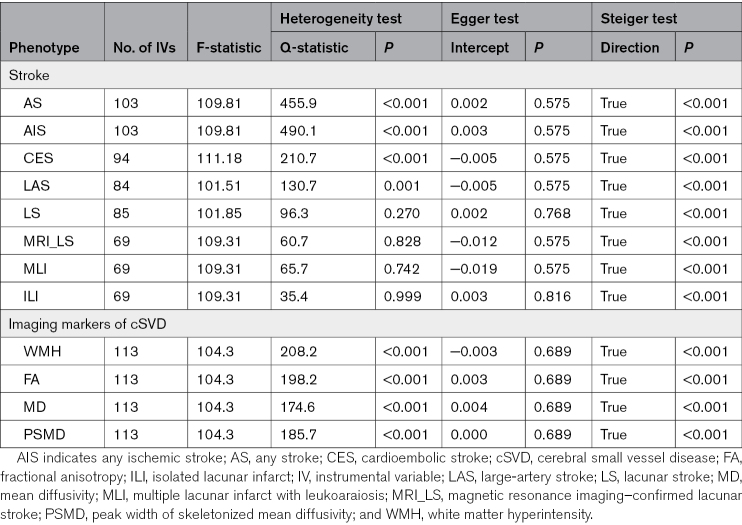
Summary Information on the Genetic Instruments for Venous Thrombosis Used in Mendelian Randomization and Sensitivity Analyses Results

### Thrombosis and Stroke

The associations between genetically predicted risk of venous thrombosis and different stroke subtypes are shown in the Figure [A] and Table S4. There was a significant association between genetically predicted risk of venous thrombosis and both AS and AIS using the inverse-variance weighted method (AS: odds ratio [OR], 1.145 [95% CI, 1.091–1.203]; *P*<0.001; AIS: OR, 1.189 [95% CI, 1.126–1.256]; *P*<0.001). However, these associations were markedly different across ischemic stroke subtypes. Using stroke outcomes from the GIGASTROKE Consortium, genetic predisposition to venous thrombosis was significantly associated with both cardioembolic and large-artery stroke (CES: OR, 1.322 [95% CI, 1.209–1.446]; *P*<0.001; LAS: OR, 1.406 [95% CI, 1.263–1.566]; *P*<0.001) but not with LS (OR, 1.074 [95% CI, 0.987–1.169]; *P*=0.156). The lack of association with LS was confirmed in the MRI-confirmed LS cohort (OR, 0.938 [95% CI, 0.809–1.088]; *P*=0.364). We also found no associations with the 2 subtypes of LS (multiple lacunar infarcts with leukoaraiosis: OR, 1.031 [95% CI, 0.842–1.263]; *P*=0.764; isolated lacunar infarct: OR, 0.927 [95% CI, 0.737–1.166]; *P*=0.593).

**Figure.**
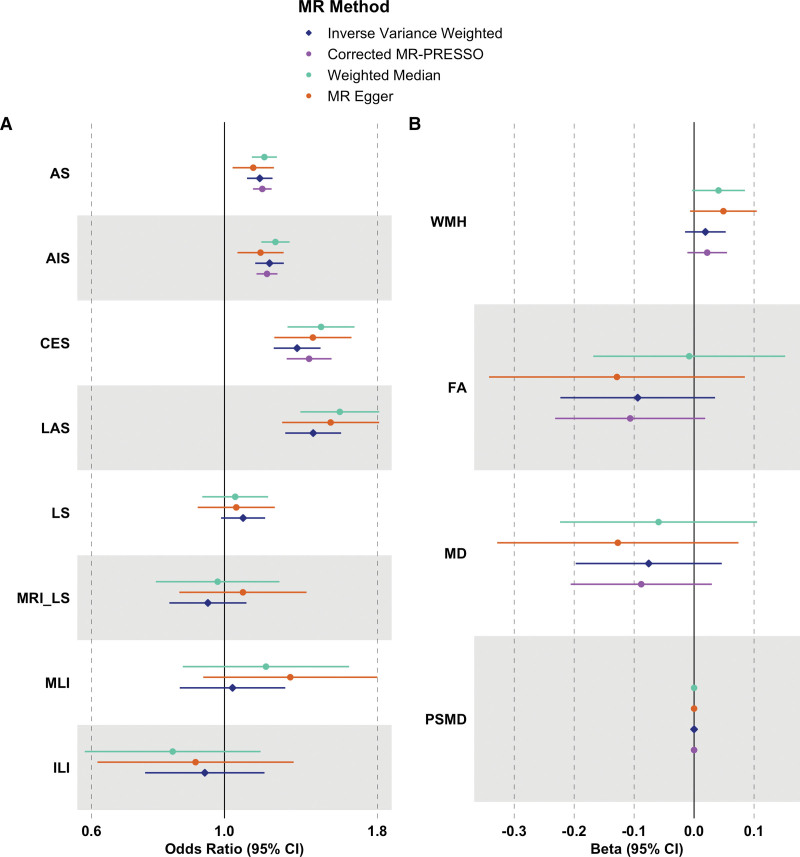
**Forest plot.** Forest plot of causal estimates and their 95% CIs between genetically predicted risk of (**A**) venous thrombosis and stroke and (**B**) imaging markers of cerebral small vessel disease (cSVD) using 2-sample Mendelian randomization (MR) methods. AIS indicates any ischemic stroke; AS, any stroke; CES, cardioembolic stroke; FA, fractional anisotropy; ILI, isolated lacunar infarct; LAS, large-artery stroke; LS, lacunar stroke; MD, mean diffusivity; MLI, multiple lacunar infarcts with leukoaraiosis; MRI_LS, magnetic resonance imaging–confirmed lacunar stroke; MR-PRESSO, Mendelian Randomization Pleiotropy Residual Sum and Outlier; PSMD, peak width of skeletonized mean diffusivity; and WMH, white matter hyperintensity.

Similar results were found using MR-Egger and the weighted median-based method. We did not identify evidence of directional pleiotropy (*P* for the Egger intercept >0.05; Table). There was some evidence of heterogeneity among variants for AS, AIS, CES, and LAS (*P*<0.05; Table), indicating that ≥1 variants serve as outliers and might have pleiotropic effects. However, the results were similar after excluding potential pleiotropic variants using the outlier-corrected MR-PRESSO (Figure [A]; Table S4; 12 variants for AS, 13 variants for AIS, and 2 variants for CES were removed). Furthermore, there was no evidence of distortion in the causal estimates resulting from inverse-variance weighted before and after the removal of outlier variants in the MR-PRESSO distortion test (Table S4). In the leave-one-out sensitivity analyses, no single instrument was strongly driving the overall effect of thrombosis on each stroke outcome. The scatter plots of the Egger regression test, funnel plots, and leave-one-out plots are also provided in Figures S1 through S3, respectively.

### Thrombosis and Imaging Markers of SVD

Genetically predicted risk of venous thrombosis was not associated with either WMH volume or DTI imaging markers of cSVD using the inverse-variance weighted method (WMH: β, 0.019 [95% CI, −0.015 to 0.053]; *P*=0.365; fractional anisotropy: β, −0.094 [95% CI, −0.223 to 0.035]; *P*=0.365; MD: β, −0.076 [95% CI, −0.197 to 0.046]; *P*=0.365; and PSMD: β, 0.000 [95% CI, 0.000–0.000]; *P*=0.540; Figure [B]; Table S5). Although there was some heterogeneity among variants (*P*<0.05; Table), no evidence of substantial horizontal pleiotropy was identified in the MR-PRESSO distortion test and the Egger regression test (*P* for the Egger intercept >0.05; Table). Furthermore, MR-Egger, the weighted median-based method, and the outlier-corrected MR-PRESSO showed consistent null results (Figure [B]; Table S5; 1 variant for WMH, 1 variant for fractional anisotropy, 1 variant for MD, and 4 variants for PSMD were removed). In the leave-one-out sensitivity analyses, there was no evidence that the overall effect of thrombosis on each imaging marker was primarily influenced by a single instrument. The scatter plots, funnel plots, and leave-one-out plots are also provided in Figures S4 through S6, respectively.

## DISCUSSION

In this study, we investigated whether thrombosis was associated with LS and imaging markers of cSVD using 2-sample MR analyses. While genetic predisposition to venous thrombosis was significantly associated with all ischemic stroke and both thromboembolic subtypes of IS, namely, CES and LAS, we found no statistically significant associations with LS or with imaging markers of cSVD. Our results were confirmed using data from a cohort with MRI-confirmed LS. Therefore, in contrast to the other stroke subtypes, our data suggest that thrombotic abnormalities do not play a causal role in LS or cSVD, a finding that may have major therapeutic implications.

The role of thrombosis in the pathogenesis of LS has been controversial. Although thrombosis has been suggested by some to play a role, thrombus has not been directly visualized. It is possible that this is due to methodological limitations because the small arterioles are difficult to visualize in vivo, and postmortem assessments often occur several years following the symptomatic vascular event due to the largely nonfatal nature of LSs.^5^ Alternatively, it may be that thrombosis is not an important initiating event. Indirect evidence from randomized controlled trials is also not conclusive. There is only one large randomized controlled trial in well-phenotyped LS. The SPS3 trial (Secondary Prevention of Small Subcortical Strokes), in over 3000 patients with MRI-confirmed LS, found that the addition of clopidogrel to aspirin did not lead to any further reduction in ischemic strokes, suggesting a lack of effect of anti-platelet agents, but did significantly increase major hemorrhage rate.^6^ No secondary stroke prevention trial comparing the efficacy of single antiplatelet versus placebo in patients with solely LS has been performed.^5^ Many reviews have tried to assess efficacy in patients with LS within larger trials including all stroke subtypes, and meta-analyses suggest efficacy for single antiplatelet agents in LS.^5,45^ However, such trials used simple stroke subtyping methods, and it has been shown that subtyping of LS based on clinical syndromes and utilizing acute CT imaging has a specificity as low as 50%, making firm conclusions difficult to draw.^22,46^

The situation is further complicated by LS itself being heterogeneous. Fisher^16^ described the pathological changes underlying LS during serial sectioning in his seminal 1969 paper as being mainly due to segmental arterial disorganization in arterioles of 40 to 200 µm in diameter. However, some specimens were observed to have solely atherosclerotic plaque at the orifice of small penetrating arteries supplying the area of infarction. These early observations have led to the hypothesis that there are 2 main vascular pathologies underlying LS: (1) hypertensive arteriopathy/arteriolosclerosis and (2) branch orifice microatheromatous disease.^5^ These have different MRI appearances with larger isolated lacunar infarcts or smaller multiple lacunar infarcts often with other features of diffuse cSVD such as confluent WMH.^17^ It has been suggested that thrombosis may be more important in the microatheromatous category.^5^ However, in this study, we found no evidence that altered thrombosis was associated with either of the 2 subtypes of LS.

A major problem in investigating a relationship such as between thrombotic factors and LS is determining causality. For example, although some studies have identified altered coagulation markers in cSVD,^47,48^ whether such alterations are merely secondary to the tissue damage itself can be difficult to determine. MR techniques provide important insights into causality. Our data, using large genetic databases, do not support thrombosis being important in LS. This is despite strong associations of both CES and LAS with a genetic predisposition to thrombosis, suggesting that our genetic instruments had sufficient sensitivity to detect any associations. By including a well-phenotyped cohort with MRI-confirmed outcomes, we were also able to confirm our findings in a cohort with higher specificity of ischemic stroke subtyping and explore associations within the 2 proposed subtypes of LS. We also found no association with more chronic markers of cSVD, WMH, or white matter ultrastructural damage on MRI. Taken together, our results demonstrate that the association with venous thrombosis differs markedly between LS and the other major stroke subtypes and suggests that altered thrombosis may not play a causal role in LS and cSVD.

Our findings have important clinical implications. cSVD is a major factor for intracerebral hemorrhage,^7^ and both dual antiplatelet therapy and anticoagulants have been associated with intracerebral hemorrhage in patients with LS and cSVD, particularly those with more severe MRI markers of cSVD. Our results suggest that more trials in well-subtyped cohorts with LS are required to evaluate the efficacy of different antithrombotic regimens in LS.

Our study has many strengths. We used summary statistics from the largest GWAS data sets for both venous thrombosis and ischemic stroke and its subtypes, so our MR study was well-powered to detect small effects. LS subtyping can be inaccurate, particularly when CT-based diagnostic algorithms are used. To provide reassurance in our findings, we replicated them in an MRI-confirmed LS cohort in which we found similar results. We further used this cohort to examine associations with subtypes of LS and also examined associations with chronic cSVD using data from GWAS from imaging markers of cSVD.

However, our study also has limitations. First, there was partial sample overlap among GWAS data sets, such as the UK Biobank data set, which was included in both the GIGASTROKE Consortium and the GWAS of VTE. This may lead to weak instrument bias in 2-sample MR.^12^ Nonetheless, the potential bias caused by sample overlap is expected to be minimal with relatively large sample sizes.^49^ Furthermore, the genetic variants selected as IVs had a high average F-statistic (up to 100) for each specific outcome, indicating that they were unlikely to be susceptible to weak instrument bias. In addition, the MRI-confirmed LS cohort had no overlap with the GWAS of VTE. Another limitation is that there was some heterogeneity among IVs for AS, AIS, and CES. However, the sensitivity analyses provided consistent estimates. Moreover, the main findings were similar before and after excluding outliers identified by the MR-PRESSO test. Furthermore, it should be noted as a limitation that LAS, CES, MRI_LS, and its subtypes showed associations with a few independent variants (r^2^<0.001), which prevented us from assessing the reverse direction of associations. However, the MR-Steiger directionality test confirmed the true direction of the causal effect of venous thrombosis on the outcomes of interest. Another limitation is that the analysis was done in a population consisting of individuals of European ancestry. More data on LS from non-European ancestries are needed to assess the generalizability of our findings to other ethnic groups. Finally, we looked at only one aspect of the thrombotic process, namely, thrombosis as assessed in the venous system. This being a relevant marker for arterial stroke is supported by prior data showing marked genetic overlap between venous thrombosis and stroke^19^ and by our results showing highly significant associations with all ischemic stroke, as well as cardioembolic and large-artery stroke subtypes. However, this does not capture all aspects of the thrombotic process and, in particular, the role of altered platelet reactivity. We were unable to examine this as there were insufficient GWAS data on this parameter to enable us to investigate this using MR.

In conclusion, our MR findings showed that venous thrombosis increases the risk of both CES and LAS stroke, which are both believed to result from thromboembolism, but it is not related to LS or imaging markers of cSVD. This raises the possibility that antithrombotic medication may be less effective in cSVD and underscores the need for further trials in well-subtyped cohorts with LS to evaluate the efficacy of different antithrombotic regimens in LS. It also highlights the importance of exploring alternative mechanisms underlying the development and progression of LS and evaluating imaging markers of cSVD that may represent potential therapeutic targets.

## ARTICLE INFORMATION

### Acknowledgments

The authors express their appreciation to the researchers, staff, and all individuals who participated in the various studies involved in this research. This study made use of the UK Biobank resource under application number 36509 and the summary statistics from the Cohorts for Heart and Aging Research in Genomic Epidemiology (CHARGE) Consortium through the application at the Database of Genotypes and Phenotypes (dbGaP), study accession number phs000930.v10.p1. The authors acknowledge the essential role of the CHARGE Consortium in the development and support of this research (see http://web.chargeconsortium.com for more details). The authors thank the investigators, the staff, and the participants of each contributing cohort in the CHARGE Consortium publication from which these results were obtained. Support for the CHARGE Consortium infrastructure was provided by the NIH grant R01 HL105756. Support for establishing and curating the dbGaP CHARGE Summary site (phs000930) was provided by the University of Virginia. All the authors contributed to the conception and design of the study. Drs Koohi, Harshfield, and Shatunov contributed to the acquisition and analysis of data. All the authors contributed to drafting the text or critically revising the article.

### Sources of Funding

This research was funded by a British Heart Foundation Program grant (RG/F/22/110052). Infrastructural support was provided by the Cambridge British Heart Foundation Centre of Research Excellence (RE/18/1/34212) and the Cambridge University Hospitals National Institute for Health and Care Research (NIHR) Biomedical Research Centre (NIHR203312). Dr Harshfield was supported by the Alzheimer’s Society (AS-RF-21-017). The views expressed are those of the authors and not necessarily those of the NIHR or the Department of Health and Social Care.

### Disclosures

None.

### Supplemental Material

STROBE-MR Checklist

Supplemental Methods

Tables S1–S5

Figures S1–S6

## Supplementary Material


