# Supplementary material for: Does Thrombosis Play a Causal Role in Lacunar Stroke and Cerebral Small Vessel Disease?
Source: Stroke. 2024 Mar 26;55(4):934–42. doi: 10.1161/STROKEAHA.123.044937 (PMC10962440; doi:10.1161/STROKEAHA.123.044937)
Supplement: Supplementary file 1 [file str-55-0934-s001.pdf]

SUPPLEMENTAL MATERIAL

Table of Contents

Supplemental Methods **2**

Description of Data for GWAS on MRI-confirmed Lacunar Stroke 2

Description of Data for GWAS on Imaging Markers of cSVD 4

Supplemental Tables **5**

Table S1. Cohort Sample Inclusion for GWAS on MRI-Confirmed Lacunar Stroke 5

Table S2. Sample Selection Description for GWAS on Imaging Markers of cSVD 6

Table S3. Instrumental Variables Used in Mendelian Randomization Analyses 7

Table S4. Association Between venous thrombosis and Stroke Using Two-Sample Mendelian Randomization Methods 11

Table S5. Association Between venous thrombosis and Imaging Markers of cSVD Using Two-Sample Mendelian Randomization 12

Supplemental Figures **13**

Figure S1. Mendelian Randomization Scatter Plots of the Effect of venous thrombosis on Stroke 13

Figure S2. Mendelian Randomization Funnel Plots of the Effect of venous thrombosis on Stroke 14

Figure S3. Leave-one-out Sensitivity Analysis Plots of the Effect of venous thrombosis on Stroke 15

Figure S4. Mendelian Randomization Scatter Plots of the Effect of venous thrombosis on Imaging Markers of cSVD 17

Figure S5. Mendelian Randomization Funnel Plots of the Effect of venous thrombosis on Imaging Markers of cSVD 18

Figure S6. Leave-one-out Sensitivity Analysis Plots of the Effect of venous thrombosis on Imaging Markers of cSVD 19

Supplemental Methods

Description of Data for GWAS on MRI-confirmed Lacunar Stroke

Cases

The stroke patients participated in the studies included in this GWAS (Supplementary Table S1) were recruited from both acute stroke admissions and outpatient services in Europe, the USA, South America, and Australia. All patients underwent a comprehensive stroke assessment, which included MRI of the brain, imaging of the carotid arteries, and an electrocardiogram. Echocardiography was performed when necessary. All clinical histories and MRIs were reviewed centrally using a standardized form to confirm the diagnosis of LS. MRI-confirmed LS was defined as a clinical lacunar syndrome, a subcortical or brain stem lesion with a diameter of less than 15 mm on MRI, and the absence of other potential sources for stroke other than cSVD (23). Patients with evidence of (1) stenosis of greater than 50% in the extracranial or intracranial cerebral vessels or previous carotid endarterectomy; (2) a cortical infarct on MRI; (3) a cardioembolic source for stroke as defined by the TOAST trial criteria (20); and (4) any other identifiable cause of stroke (e.g., lupus anticoagulant, cerebral vasculitis, dissection, or monogenic causes of stroke) were excluded. For the current study, we focused exclusively on cases and controls of European ancestry.

Controls

The majority of controls in our study were indeed drawn from the following studies:

The NINDS-SIGN (National Institute of Neurological Disorders and Stroke - Stroke Genetics Network) includes ischemic stroke cases from 24 genetic research canters: 13 from the United States and 11 from Europe. Whenever feasible, controls with publicly available genotype data were selected to ancestry-match cases at each genetic research centre. A key factor in the selection process for these control groups was their previous genotyping using the Illumina Omni series GWAS array to minimize potential technical discrepancies between cases and controls (For more details on these studies, please see: *https://www.ahajournals.org/doi/epub/10.1161/STROKEAHA.113.001857).*

The Understanding Society, also known as United Kingdom Household Longitudinal Study (UKHLS), is a comprehensive longitudinal panel survey that involves 40,000 households across the United Kingdom (including England, Scotland, Wales, and Northern Ireland). These households were chosen to be representative of the UK population. Out of these individuals, 10,484 samples were genotyped at the Wellcome Trust Sanger Institute, using the Illumina Infinium HumanCoreExome-12 v1.0BeadChip (For more information, please see: *https://www.understandingsociety.ac.uk*).

The Young Lacunar Stroke DNA Resource (DNA Lacunar 1) recruited a total of 1,029 Caucasian patients with lacunar stroke, aged ≤70 years from 72 specialist's stroke centres across the UK during 2002-2012. Unrelated white individuals without any clinical cerebrovascular disease were selected as controls from general practice lists in the same region as the patients through random sampling, stratified for age and sex. A standardized clinical evaluation and a standardized study questionnaire were completed by all patients and controls. MRI wasn't used on the controls.

The Wellcome Trust Case-Control Consortium 2 (WTCCC2) recruited stroke cases from three centers in the UK (St. George's University London, Oxford, and Edinburgh) and one center in

Germany (University and Klinikum Großhadern, Ludwig-Maximilians-University, Munich). Controls for the UK samples were drawn from shared WTCCC controls obtained from the 1958 Birth Cohort, a prospectively collected cohort of individuals born in 1958 and part of the national child development study. For the German samples, controls were Caucasians of German origin participating in the population KORAgen study, which represents a gender- and age-stratified random sample of all German residents in the Augsburg area. All controls had no history of stroke or transient ischemic attack.

Genetic quality control

For the genetic quality control, variants with a MAF < 1%, a missing rate per SNP > 3%, a missing rate per individual > 5%, an imputation RSQ score <0.3, a Hardy-Weinberg disequilibrium p-value $\leq 1 \times 10^{-6}$ or monomorphic (A/T, C/G) variants were excluded from analysis. To control inflation factor for estimated statistics due to population structure, we conducted a principal component analysis (PCA) on LD-pruned dataset, as implemented in plink2, and used the first six PCAs as covariates in logistic regression model.

Description of Data for GWAS on Imaging Markers of cSVD

We utilized image-derived phenotypes provided by UK Biobank, including (1) total volume of WMH derived from T1 and T2_FLAIR images (WMH, field 25781), which we log-transformed and normalized for brain volume (field 25009), (2) FA (fields 25056-25103), and (3) MD (fields 25104-25151). Individuals whose imaging markers were outside ±6 SD of the mean were excluded. To obtain a single global measure for FA and MD from the DTI images, we performed PCA, as a dimension reduction method, on the FA and MD markers of each of the 48 individual white matter regions using FactoMineR, and the first principal component for FA and MD was used for association analysis.

Individuals with single gene mutations causing cSVD in the NOTCH3, HTRA1, or COL4A1/2 loci, or diagnosed with multiple sclerosis, were excluded from the analysis. Based on the sample QC description provided by the UK Biobank, we also excluded (1) related individuals with a kinship coefficient of ≥ 0.0884, ensuring only one person was retained from each group with up to second-degree relationships; (2) individuals with a mismatch between self-reported and genotypic sex; (3) outliers identified based on measures of heterozygosity and genotype missingness; (4) those with a missing rate > 5%; (5) individuals not of European ancestry; and (6) individuals whose first population PCAs were outside the ±6 SD range.

For quality control, we excluded variants with a MAF < 1%, a missing rate per SNP > 10%, a missing rate per individual > 10%, or a Hardy-Weinberg disequilibrium p-value ≤ 1 × 10−15.

Supplemental Tables

Table S1. Cohort Sample Inclusion for GWAS on MRI-Confirmed Lacunar Stroke

Study	Cases					Controls		
	LS	MLI	ILI	% Male	Mean Age (SD)	N	% Male	Mean Age (SD)
DNA Lacunar 2	986	621	362	66	66(12)	-	-	-
DNA Lacunar 1	953	465	487	71	56(9)	976	53	60(4)
NINDS-SIGN consortium	599	138	114	66	62(11)	9599	49	62(17)
SGUL Stroke Register, GENESIS	264	174	71	62	68(11)	-	-	-
WTCCC2 (UK & Germany)	195	140	54	59	69(11)	162	55	NA*
PRESERVE	45	45	0	62	68(9)	-	-	-
Edinburgh Mild Stroke Study	34	21	12	58	66(11)	-	-	-
Massachusetts General Hospital	32	18	14	72	66(15)	-	-	-
Apathy after Stroke Study	31	0	0	61	69(13)	-	-	-
Leuven Stroke Genetics Study	31	21	8	58	70(12)	-	-	-
Australian Stroke Genetics Collaborative	21	12	6	52	65(11)	25	44	-
Milano	8	3	5	75	53(16)	-	-	-
Understanding Society	-	-	-	-	-	9193	44	NA*
**Total samples for GWAS analysis**	**3,199**	**1,658**	**1,133**	**66**	**63(12)**	**19,955**	**49**	**-**

LS: lacunar stroke; MLI: multiple lacunar infarct with leukoaraiosis; ILI: isolated lacunar infarct.

* age not available in WTCCC2 and Understanding Society controls.

These studies have been described in the previous ISGC LS GWAS (Traylor M, Persyn E, Tomppo L, Klasson S, Abedi V, Bakker MK, et al. Genetic basis of lacunar stroke: a pooled analysis of individual patient data and genome-wide association studies. Lancet Neurol. 2021 May;20(5):351–61.).

Table S2. Sample Selection Description for GWAS on Imaging Markers of cSVD

	**WMH**	**PSMD**	**FA**	**MD**
Individuals with phenotype data	45,204	44,221	44,317	44,317
With multiple sclerosis	170	168	168	168
With single gene mutations causing SVD	167	165	166	166
Outliers	0	20	198	738
Total of removed samples	335	351	530	1,070
Remaining samples	44,869	43,870	43,787	43,247
No QC information	1,178	1,138	1,137	1,121
Remaining samples	43,691	42,732	42,650	42,126
Kinship >= 0.0884	476	466	464	459
Gender mismatch	23	22	22	21
Outliers (het. missing.)	45	45	45	45
Missing rate > 0.05	25	25	24	25
No European ancestry	5,726	5,600	5,594	5,525
Outliers with PCA	101	100	100	97
Total of removed samples	6,336	6,199	6,190	6,114
**Total UK Biobank samples for GWAS analysis**	**37,355**	**36,533**	**36,460**	**36,012**
**Total CHARGE consortium sample for meta-analysis**	**17,936**	**-**	**-**	**-**
**Overall total**	**55,291**	**36,533**	**36,460**	**36,012**

Table S3. Instrumental Variables Used in Mendelian Randomization Analyses

SNP	CHR	BP	EF	RF	Beta	SE	P-value
rs687621	9	136 137 065	A	G	-0.2489	0.0061	1.00e-200
rs114101204	1	169 026 054	A	G	0.5580	0.0158	1.00e-200
rs2289252	4	187 207 381	T	C	0.1623	0.0054	3.54e-196
rs2066864	4	155 525 695	A	G	0.1760	0.0060	1.06e-190
rs78707713	10	71 245 276	T	C	0.1773	0.0084	1.60e-99
rs6060308	20	33 794 378	A	G	0.0959	0.0059	5.19e-59
rs113079063	1	169 031 755	T	G	0.2678	0.0169	2.37e-56
rs116120384	1	168 910 669	A	C	0.2760	0.0187	2.42e-49
rs2360742	19	10 740 574	T	C	-0.0936	0.0068	1.83e-43
rs141325867	11	47 443 654	T	C	0.3094	0.0228	6.03e-42
rs3094326	9	136 343 647	C	G	-0.0909	0.0073	9.25e-36
rs3136516	11	46 760 756	A	G	-0.0704	0.0057	6.76e-35
rs10886430	10	121 010 256	A	G	-0.1025	0.0087	6.14e-32
rs1799809	2	128 175 875	A	G	-0.0631	0.0054	1.93e-31
rs76829066	1	170 178 132	T	G	0.1548	0.0133	2.55e-31
rs12445050	16	81 870 969	T	C	0.0896	0.0077	3.34e-31
rs6993770	8	106 581 528	A	T	0.0701	0.0062	8.31e-30
rs3211752	13	113 787 459	A	G	-0.0593	0.0054	1.28e-27
rs7135039	12	6 160 614	T	C	0.0612	0.0056	4.60e-28
rs6132574	20	23 175 922	T	G	0.0711	0.0071	1.46e-23
rs1048483	17	1 966 457	T	C	0.0506	0.0054	6.60e-21
rs174566	11	61 592 362	A	G	0.0513	0.0056	5.97e-20
rs139541321	1	169 784 737	T	C	0.1476	0.0164	2.50e-19
rs1867312	2	68 619 981	A	C	-0.0485	0.0054	2.11e-19
rs892090	19	55 539 072	T	G	-0.0667	0.0075	3.57e-19
rs78667607	1	207 271 862	T	G	-0.1144	0.0130	1.37e-18
rs1884841	14	92 309 229	A	G	0.0470	0.0054	3.26e-18
rs141798115	11	56 875 074	T	C	0.2036	0.0237	9.40e-18
rs6795524	3	93 650 604	A	G	-0.1421	0.0168	2.93e-17
rs2844543	6	31 347 204	A	T	0.0467	0.0056	3.98e-17
rs62350309	4	187 277 666	A	G	0.0924	0.0112	1.20e-16
rs55823018	16	88 535 242	T	C	-0.0483	0.0059	2.29e-16
rs34377151	10	71 202 996	T	C	0.0671	0.0086	6.07e-15
rs55747751	5	132 397 351	A	G	-0.0732	0.0096	2.62e-14

SNP	CHR	BP	EF	RF	Beta	SE	P-value
rs13055886	22	43 104 750	T	C	0.0448	0.0059	2.35e-14
rs214060	6	25 537 194	T	C	0.0408	0.0054	4.85e-14
rs72707947	1	168 609 132	T	G	-0.1122	0.0149	4.14e-14
rs112901519	11	56 273 318	T	G	0.1014	0.0135	6.57e-14
rs12934767	16	75 456 304	T	C	0.0443	0.0059	3.58e-14
rs10181664	2	36 783 215	T	C	0.0435	0.0058	4.90e-14
rs140602438	3	90 067 332	C	G	0.1403	0.0188	8.38e-14
rs11534419	9	136 388 223	T	C	-0.0417	0.0056	1.43e-13
rs4759076	12	54 729 872	T	C	0.0422	0.0057	1.98e-13
rs142316601	4	187 069 574	T	G	-0.1527	0.0207	1.51e-13
rs28929474	14	94 844 947	T	C	0.1348	0.0184	2.38e-13
rs141622900	19	45 426 792	A	G	0.0877	0.0120	2.33e-13
rs4002471	19	49 215 095	T	C	0.0393	0.0054	4.22e-13
rs5030062	3	186 454 180	A	C	-0.0398	0.0055	6.38e-13
rs3001023	1	201 768 789	A	G	-0.0397	0.0055	6.39e-13
rs9373523	6	147 701 133	T	G	-0.0389	0.0054	8.44e-13
rs77542162	17	67 081 278	A	G	-0.1293	0.0180	6.95e-13
rs597808	12	111 973 358	A	G	0.0385	0.0054	9.22e-13
rs10087301	8	27 820 792	A	G	0.0493	0.0070	1.36e-12
rs12887044	14	75 255 659	T	C	0.0406	0.0058	2.98e-12
rs7948396	11	49 124 908	T	G	-0.0374	0.0054	6.48e-12
rs6036192	20	22 703 677	T	C	0.0411	0.0060	1.02e-11
rs9854955	3	156 795 525	A	G	0.0383	0.0056	6.12e-12
rs117653193	11	50 242 788	T	C	0.0657	0.0097	1.51e-11
rs34290760	8	9 185 179	C	G	-0.0994	0.0147	1.48e-11
rs57615042	1	218 699 815	A	G	-0.0552	0.0082	1.99e-11
rs139974673	15	44 027 885	T	C	0.1253	0.0187	1.83e-11
rs117273915	11	55 166 020	T	G	-0.0690	0.0103	2.25e-11
rs56324901	3	194 788 252	A	G	-0.0447	0.0067	3.11e-11
rs67032674	20	32 725 947	A	G	-0.0735	0.0112	4.97e-11
rs35257264	11	126 296 816	T	C	0.1094	0.0167	5.34e-11
rs185699757	12	6 071 752	A	G	-0.0809	0.0124	6.36e-11
rs3811444	1	248 039 451	T	C	-0.0370	0.0057	5.73e-11
rs4759420	12	123 794 871	C	G	0.0427	0.0066	1.13e-10
rs11774252	8	6 651 969	A	G	-0.0368	0.0057	9.35e-11
rs3751198	12	104 147 207	A	G	-0.0355	0.0055	1.20e-10

SNP	CHR	BP	EF	RF	Beta	SE	P-value
rs10516757	4	86 704 797	A	G	0.0527	0.0082	1.25e-10
rs9872742	3	126 273 130	A	G	-0.0392	0.0061	1.08e-10
rs11672660	19	46 180 184	T	C	-0.0420	0.0066	1.74e-10
rs7188250	16	53 834 607	T	C	-0.0343	0.0054	2.59e-10
rs1190982	14	58 815 839	T	C	-0.0366	0.0058	2.90e-10
rs6137837	20	23 108 362	A	C	-0.0359	0.0057	2.95e-10
rs42038	7	92 243 719	T	C	0.0362	0.0058	6.17e-10
rs1047891	2	211 540 507	A	C	0.0361	0.0058	3.80e-10
rs1173727	5	32 830 521	T	C	0.0336	0.0054	5.62e-10
rs17502085	15	96 125 226	A	G	0.0391	0.0063	4.68e-10
rs33944211	3	93 768 268	C	G	0.0557	0.0090	5.29e-10
rs3823363	6	29 943 715	T	C	-0.0346	0.0056	6.37e-10
rs4889599	16	30 968 589	T	C	-0.0343	0.0056	7.77e-10
rs12517759	5	38 709 629	A	G	0.0361	0.0059	1.22e-09
rs738408	22	44 324 730	T	C	-0.0396	0.0065	1.12e-09
rs1363976	5	96 248 795	T	C	-0.0347	0.0057	1.05e-09
rs713996	22	33 177 778	A	G	-0.0334	0.0055	1.56e-09
rs6693697	1	150 264 106	T	C	0.0382	0.0063	1.62e-09
rs10990535	9	99 091 009	T	C	-0.0377	0.0063	1.92e-09
rs375178383	11	48 737 651	T	C	0.1495	0.0250	2.34e-09
rs12898055	14	103 835 290	A	C	-0.0346	0.0058	1.81e-09
rs2978457	8	42 324 760	T	C	0.0344	0.0058	2.14e-09
rs1070073	12	104 000 319	T	G	-0.0340	0.0058	4.67e-09
rs78624203	1	168 378 408	A	G	0.0949	0.0162	4.40e-09
rs11044893	12	19 994 917	C	G	-0.0561	0.0096	4.89e-09
rs7905967	10	21 873 295	T	G	0.0356	0.0061	4.62e-09
rs9264579	6	31 235 746	A	G	-0.0350	0.0060	7.30e-09
rs1870940	1	154 984 363	A	G	0.0348	0.0060	7.14e-09
rs10993706	9	93 602 967	A	G	0.0603	0.0104	6.10e-09
rs3754120	1	118 146 031	T	G	-0.0677	0.0117	8.20e-09
rs28752523	6	32 584 772	T	C	-0.0419	0.0073	8.85e-09
rs60509203	11	77 641 359	A	G	-0.0489	0.0086	1.39e-08
rs7069316	10	96 000 282	A	G	0.0312	0.0055	1.04e-08
rs61804164	1	161 623 025	C	G	0.0474	0.0084	1.99e-08
rs12097293	1	11 197 467	A	G	-0.0406	0.0072	1.57e-08
rs889269	5	108 165 445	T	C	-0.0388	0.0069	1.99e-08

SNP	CHR	BP	EF	RF	Beta	SE	P-value
rs17807204	17	7 784 295	A	G	-0.0584	0.0104	2.02e-08
rs1513275	7	28 259 233	T	C	0.0387	0.0069	2.58e-08
rs5757675	22	39 838 892	T	G	-0.0342	0.0061	2.10e-08
rs7960840	12	71 121 027	A	T	-0.0347	0.0062	2.41e-08
rs7744054	6	121 863 829	T	G	-0.0301	0.0054	2.75e-08
rs11054402	12	11 791 029	T	C	0.0317	0.0057	2.70e-08
rs10033399	4	83 939 171	T	C	-0.0428	0.0077	2.69e-08
rs67890964	16	83 979 317	T	C	-0.0304	0.0055	3.47e-08
rs2087072	1	243 829 549	A	G	-0.0314	0.0057	4.90e-08
rs1054533	19	17 004 049	T	C	0.0313	0.0057	4.52e-08
rs78872368	2	198 545 250	C	G	-0.0448	0.0082	4.32e-08
rs1805081	18	21 140 432	T	C	0.0295	0.0054	4.98e-08
rs59985551	2	56 106 928	T	C	-0.0355	0.0065	3.94e-08

Table S4. Association Between venous thrombosis and Stroke Using Two-Sample Mendelian Randomization Methods

	Inverse Variance Weighted		MR Egger		Weighted Median		Corrected MR-PRESSO		
**Phenotype**	**OR (95% CI)**	**P**	**OR (95% CI)**	**P**	**OR (95% CI)**	**P**	**OR (95% CI)**	**P**	**P***
**AS**	1.145 (1.091-1.203)	<0.001	1.117 (1.032-1.209)	0.015	1.166 (1.111-1.223)	<0.001	1.157 (1.116-1.199)	<0.001	0.449
**AIS**	1.189 (1.126-1.256)	<0.001	1.149 (1.051-1.255)	0.008	1.216 (1.152-1.284)	<0.001	1.178 (1.131-1.226)	<0.001	0.555
**CES**	1.322 (1.209-1.446)	<0.001	1.405 (1.211-1.629)	<0.001	1.450 (1.275-1.649)	<0.001	1.384 (1.270-1.509)	<0.001	0.456
**LAS**	1.406 (1.263-1.566)	<0.001	1.504 (1.248-1.812)	<0.001	1.558 (1.339-1.813)	<0.001	-	-	-
**LS**	1.074 (0.987-1.169)	0.156	1.046 (0.902-1.214)	0.571	1.042 (0.918-1.183)	0.598	-	-	-
**MRI_LS**	0.938 (0.809-1.088)	0.533	1.074 (0.841-1.371)	0.571	0.974 (0.768-1.235)	0.829	-	-	-
**MLI**	1.031 (0.842-1.263)	0.764	1.288 (0.922-1.799)	0.229	1.173 (0.852-1.614)	0.438	-	-	-
**ILI**	0.927 (0.737-1.166)	0.593	0.895 (0.614-1.305)	0.571	0.820 (0.585-1.149)	0.397	-	-	-

Corrected MR-PRESSO: outlier-corrected Mendelian randomization pleiotropy residual sum and outlier test; OR: odd ratio; CI: confidence interval; P; false discovery rate adjusted p-value; AS: any stroke; AIS: any ischemic stroke; CES: cardioembolic stroke; LAS: large artery stroke; LS: lacunar stroke; MRI_LS: MRI-confirmed lacunar stroke; MLI: multiple lacunar infarct with leukoaraiosis; ILI: isolated lacunar infarct.

* MR-PRESSO distortion test at P-value < 0.05.

Table S5. Association Between venous thrombosis and Imaging Markers of cSVD Using Two-Sample Mendelian Randomization

	Inverse Variance Weighted		MR Egger		Weighted Median		Corrected MR-PRESSO		
Phenotype	B (95% CI)	P	B (95% CI)	P	B (95% CI)	P	B (95% CI)	P	P*
**WMH**	0.019 (-0.015-0.053)	0.365	0.049 (-0.007-0.104)	0.320	0.041 (0.003-0.085)	0.278	0.018 (-0.008-0.045)	0.242	0.811
**FA**	-0.094 (-0.223-0.035)	0.365	-0.129 (-0.342-0.085)	0.320	-0.008 (-0.171-0.155)	0.922	-0.107 (-0.232-0.019)	0.242	0.853
**MD**	-0.076 (-0.197-0.046)	0.365	-0.127 (-0.328-0.074)	0.320	-0.059 (-0.219-0.100)	0.922	-0.088 (-0.206-0.030)	0.242	0.845
**PSMD**	0.000 (0.000-0.000)	0.540	0.000 (0.000-0.000)	0.819	0.000 (0.000-0.000)	0.922	0.000 (0.000-0.000)	0.543	0.871

cSVD: cerebral small vessel disease; Corrected MR-PRESSO: outlier-corrected Mendelian randomization pleiotropy residual sum and outlier test; B: beta coefficient; CI: confidence interval; P; false discovery rate adjusted p-value; WMH: White Matter Hyperintensities; FA: Fractional Anisotropy; MD: Mean Diffusivity; PSMD: Peak width of Skeletonised Mean Diffusivity.

* MR-PRESSO distortion test at P-value < 0.05.

Supplemental Figures

Figure S1. Mendelian Randomization Scatter Plots of the Effect of venous thrombosis on Stroke

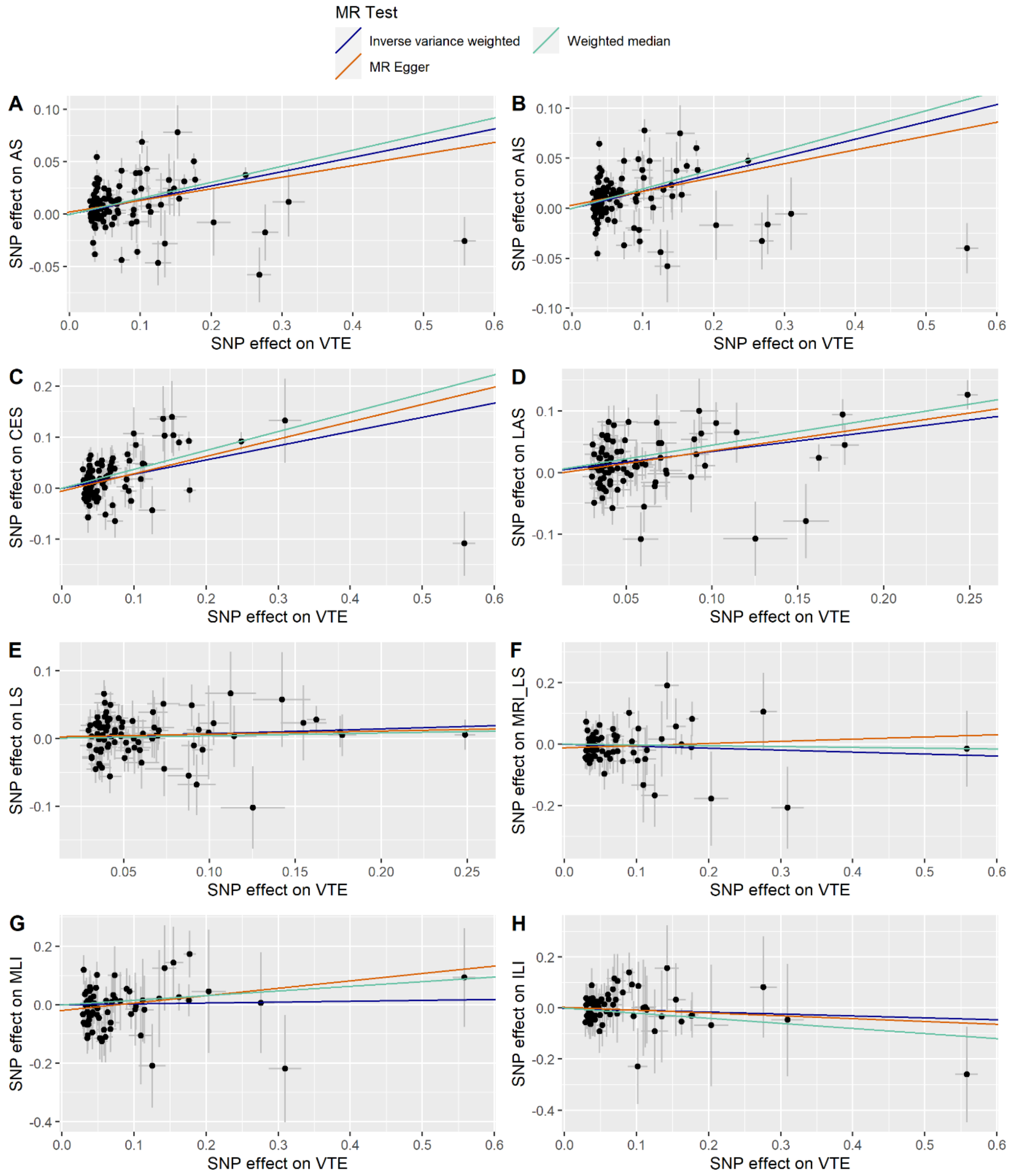

Scatter plots relating the effect sizes of the SNP- venous thrombosis association (x-axis, log OR) and the SNP-stroke associations (y-axis, log OR) with standard error bars for any stroke (**A**), any ischemic stroke (**B**), cardioembolic stroke (**C**), large artery stroke (**D**), lacunar stroke (**E**), MRI-confirmed lacunar stroke (**F**), multiple lacunar infarct with leukoaraiosis (**G**), and isolated lacunar infarct (**H**). The slopes of the lines correspond to causal estimates using each of the three different MR methods.

Figure S2. Mendelian Randomization Funnel Plots of the Effect of venous thrombosis on Stroke

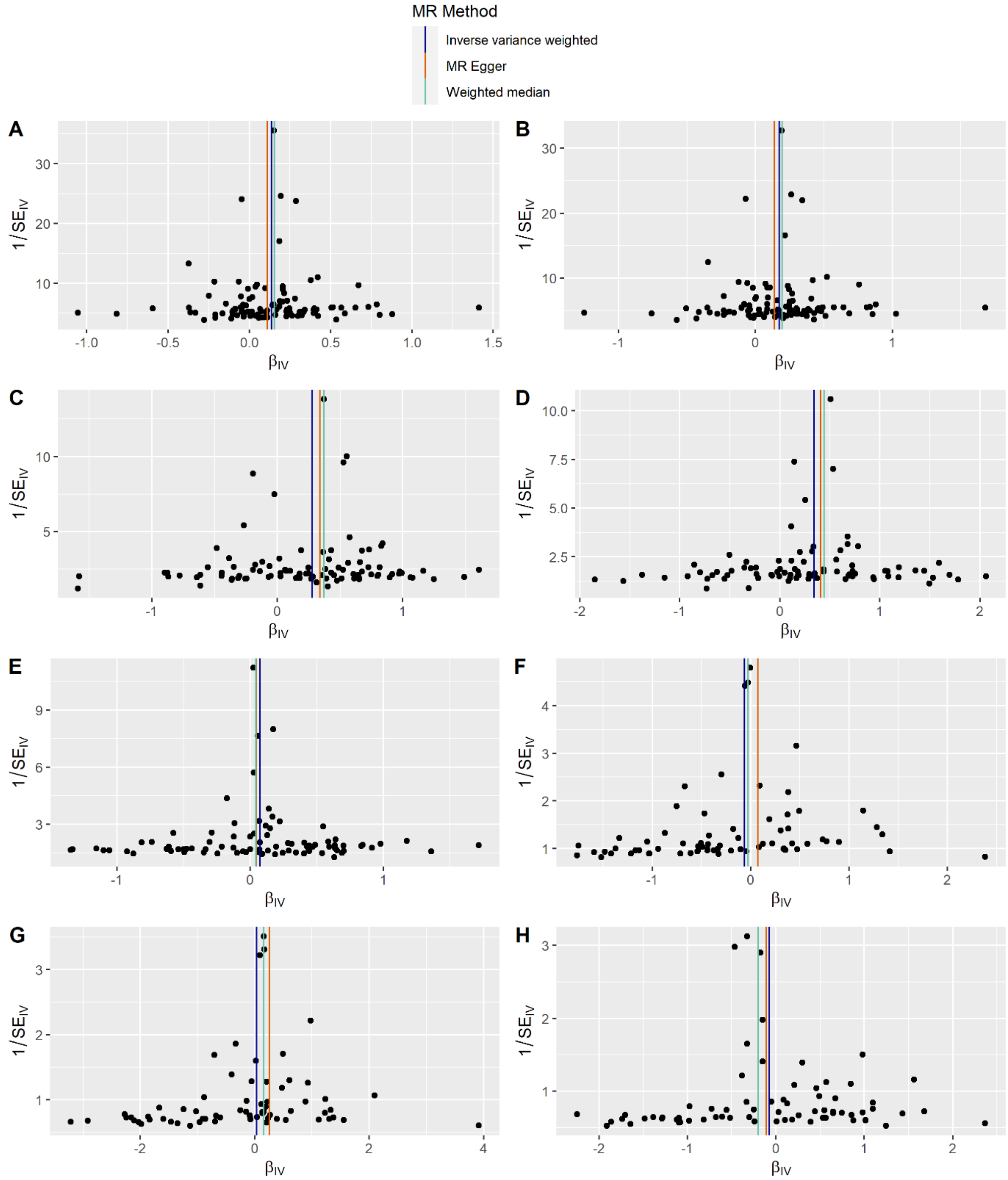

Funnel plots showing the relationship between the causal effect of venous thrombosis on any stroke (A), any ischemic stroke (B), cardioembolic stroke (C), large artery stroke (D), lacunar stroke (E), MRI-confirmed lacunar stroke (F), multiple lacunar infarct with leukoaraiosis (G), and isolated lacunar infarct (H), estimated using each individual SNP as a separate instrument against the inverse of the standard error of the causal estimate. Vertical lines show the causal estimates using all SNPs combined into a single instrument for each of three different methods.

Figure S3. Leave-one-out Sensitivity Analysis Plots of the Effect of venous thrombosis on Stroke

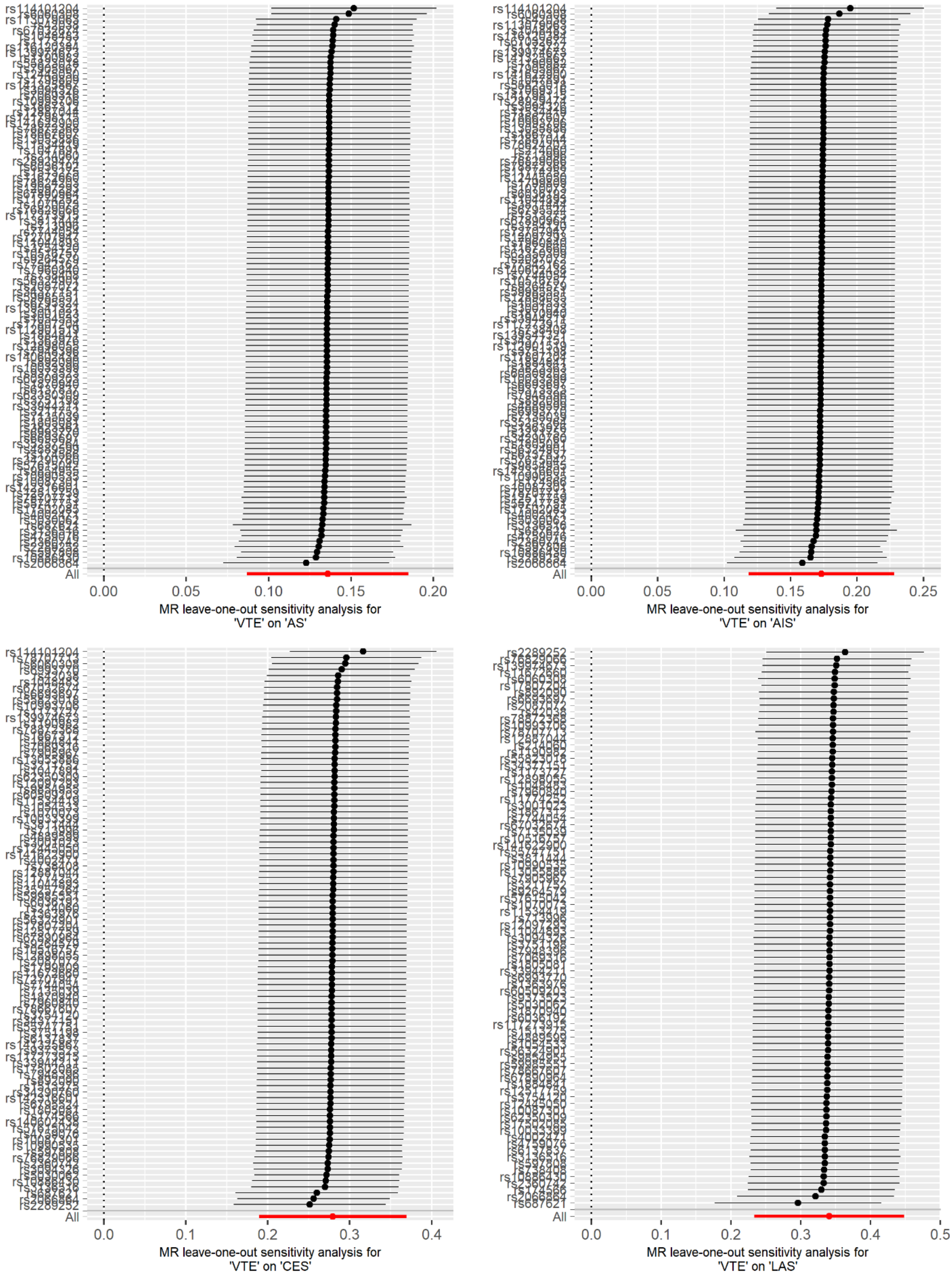

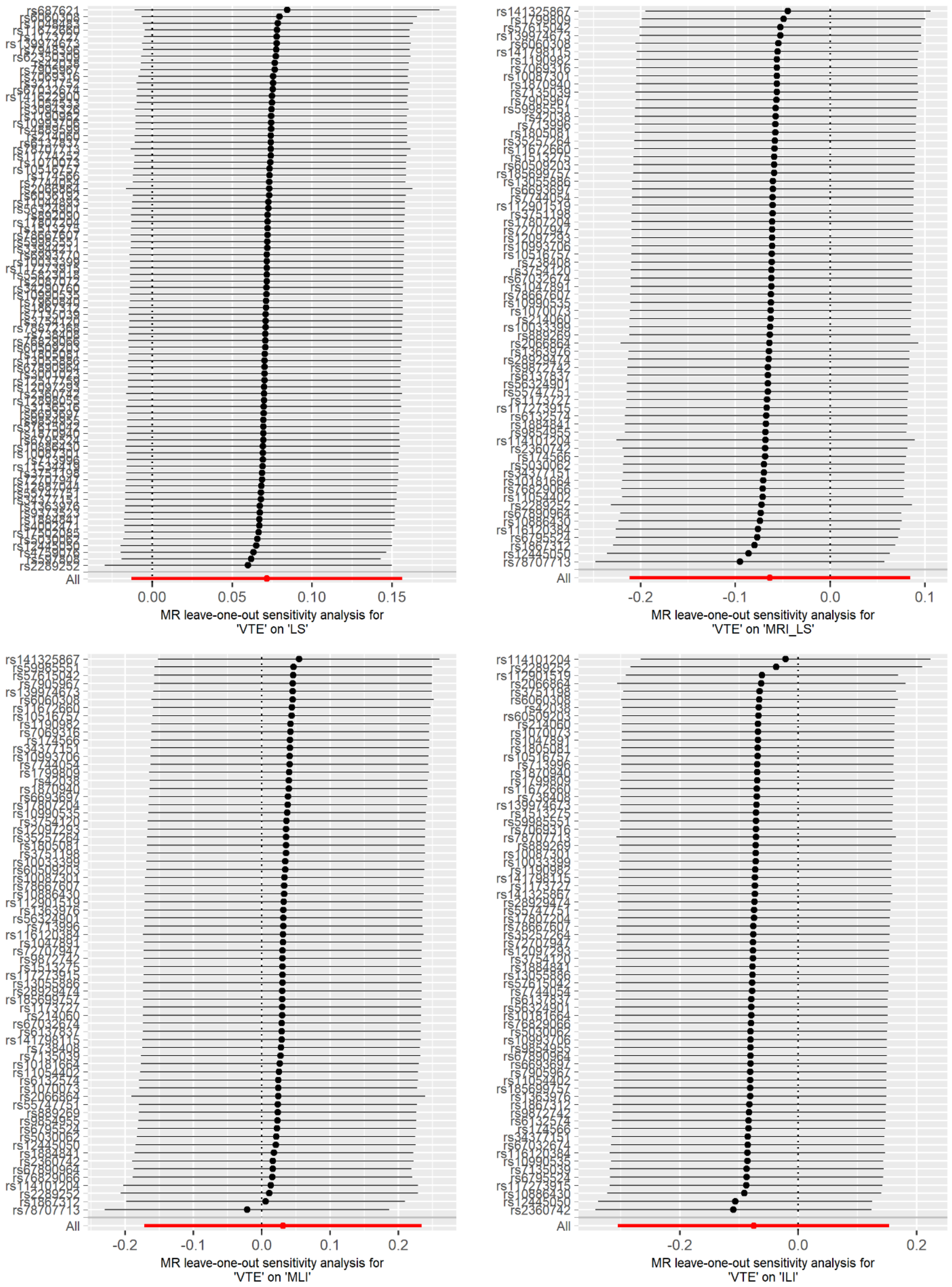

Each black point represents the log odds ratio (OR) for the causal effect of VTE on ischaemic stroke subtypes estimated using the IVW MR method by excluding that particular variant from the analysis. The red point depicts the IVW estimate (log OR) using all SNPs.

Figure S4. Mendelian Randomization Scatter Plots of the Effect of venous thrombosis on Imaging Markers of cSVD

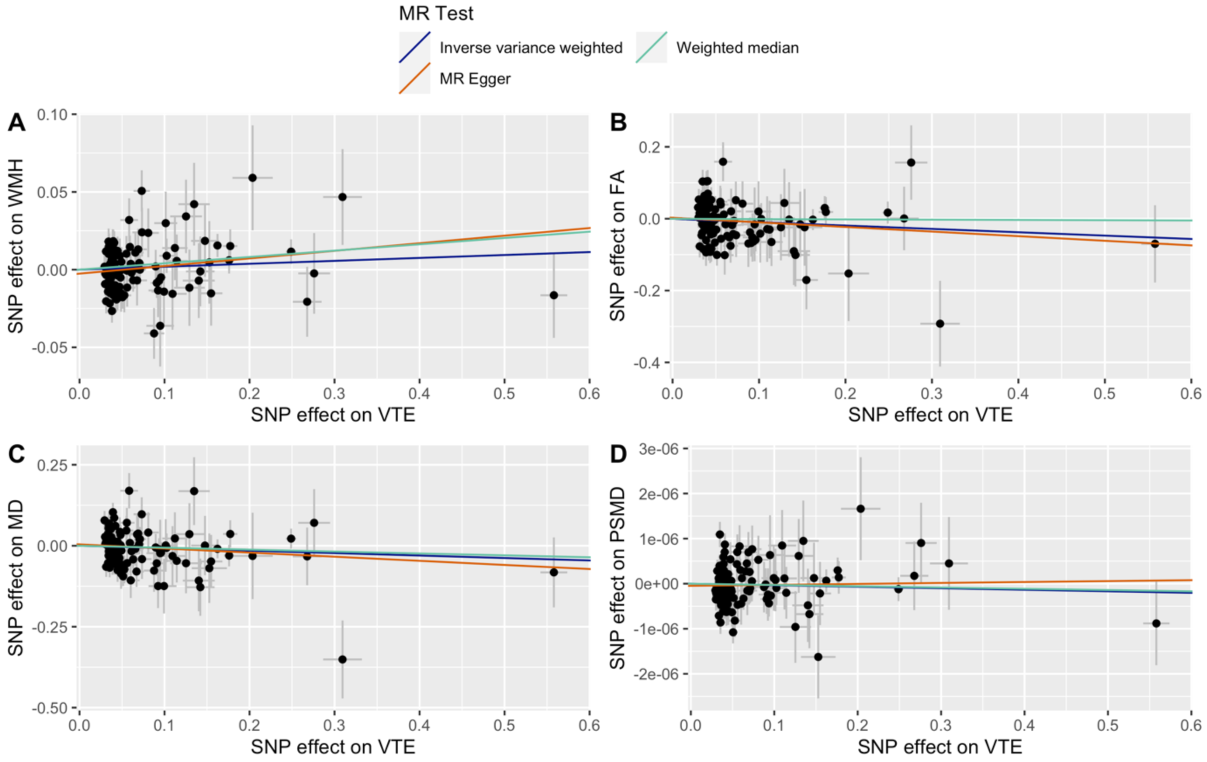

Scatter plots relating the effect sizes of the SNP- venous thrombosis association (x-axis, log OR) and the SNP-imaging marker associations (y-axis, log OR) with standard error bars for white matter hyperintensities (**A**), fractional anisotropy (**B**), mean diffusivity (**C**), and peak width of skeletonised mean diffusivity (**D**). The slopes of the lines correspond to causal estimates using each of the three different MR methods.

Figure S5. Mendelian Randomization Funnel Plots of the Effect of venous thrombosis on Imaging Markers of cSVD

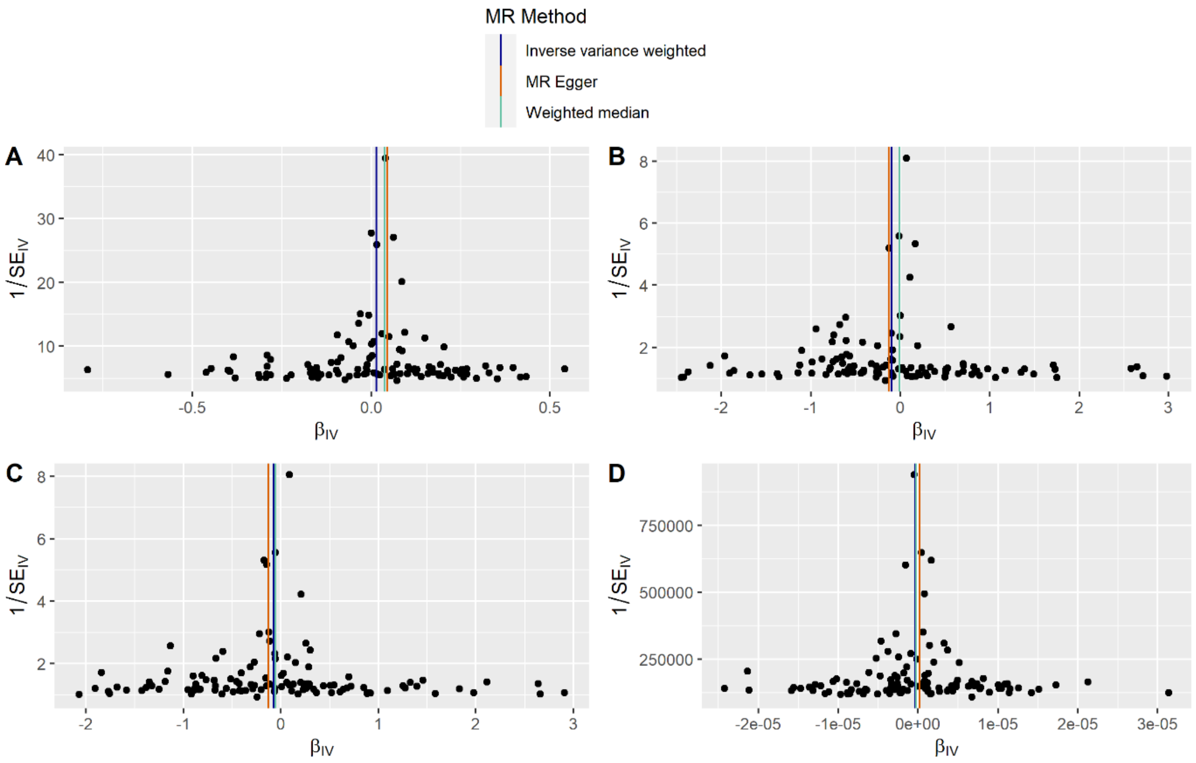

Funnel plots showing the relationship between the causal effect of venous thrombosis on white matter hyperintensities (**A**), fractional anisotropy (**B**), mean diffusivity (**C**), and peak width of skeletonised mean diffusivity (**D**), estimated using each individual SNP as a separate instrument against the inverse of the standard error of the causal estimate. Vertical lines show the causal estimates using all SNPs combined into a single instrument for each of three different methods.

Figure S6. Leave-one-out Sensitivity Analysis Plots of the Effect of venous thrombosis on Imaging Markers of cSVD

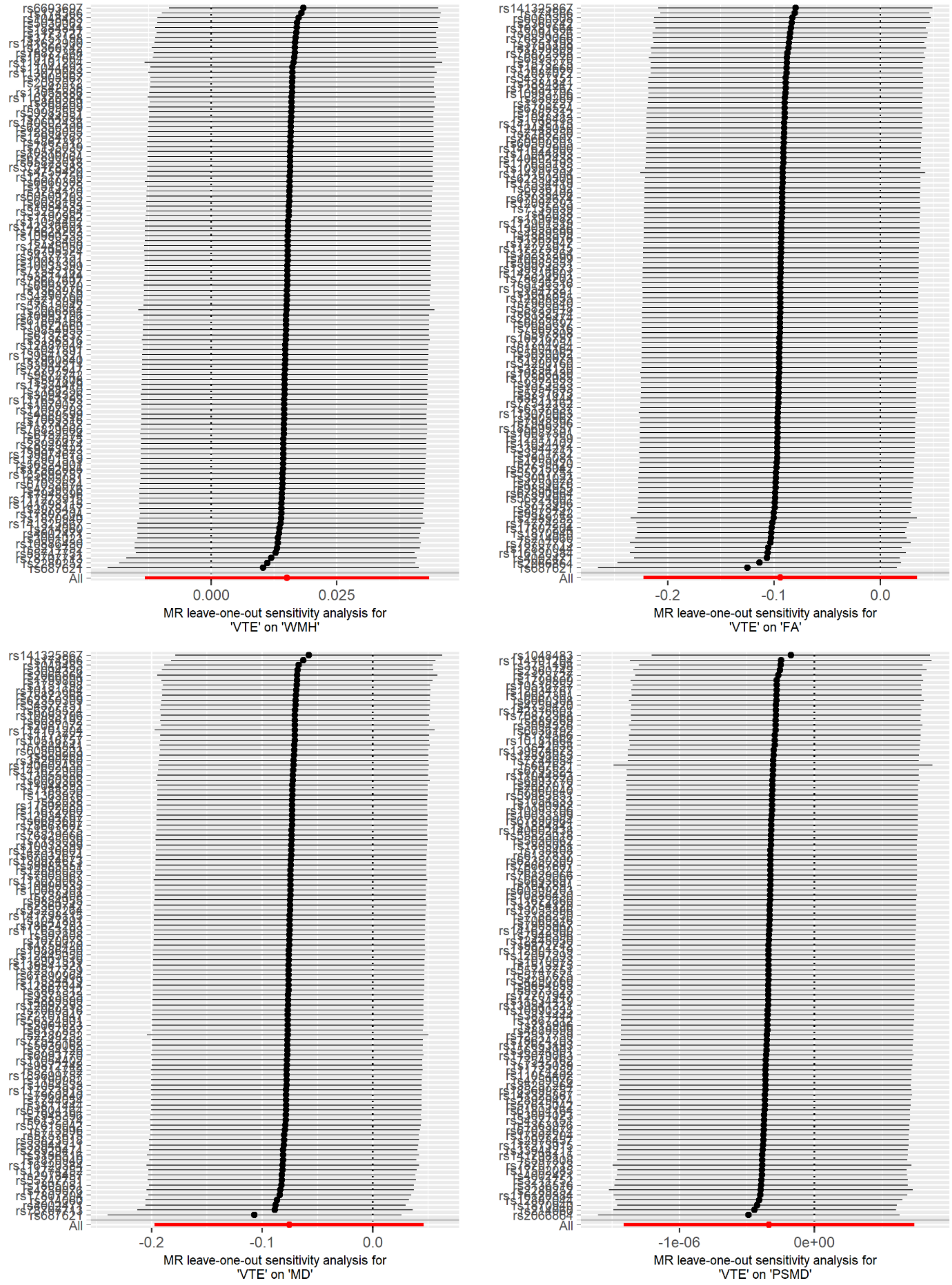

Each black point represents the log odds ratio (OR) for the causal effect of VTE on ischaemic stroke subtypes estimated using the IVW MR method by excluding that particular variant from the analysis. The red point depicts the IVW estimate (log OR) using all SNPs.
